# Molecular Survey of *Babesia* spp. and *Anaplasma phagocytophilum* in Roe Deer from a Wildlife Rescue Center in Italy

**DOI:** 10.3390/ani11113335

**Published:** 2021-11-22

**Authors:** Alessandra Cafiso, Chiara Bazzocchi, Martina Cavagna, Elena Di Lorenzo, Valentina Serra, Riccardo Rossi, Stefano Comazzi

**Affiliations:** 1 Department of Veterinary Medicine, University of Milan, 26900 Lodi, Italy; chiara.bazzocchi@unimi.it (C.B.); marti.cavagna7@gmail.com (M.C.); elena.dilo@gmail.com (E.D.L.); valentina.serra@unimi.it (V.S.); stefano.comazzi@unimi.it (S.C.); 2 Piacenza Wildlife Rescue Center, 29120 Niviano di Rivergano, Italy; info@piacenzawildlife.org

**Keywords:** *Babesia*, *Anaplasma phagocytophilum*, rescued animal, roe deer, zoonosis, tick-borne pathogen, wildlife, northern Italy, rescued animal

## Abstract

**Simple Summary:**

Roe deer, whose populations are increasing and expanding throughout Europe, are suitable hosts for the maintenance of a variety of infectious tick-vectored microorganisms, that can infect both animals and humans. In this study the presence of tick-transmitted pathogens was investigated in roe deer recovered by a wildlife rescue center based in Italy. This kind of samples represents a convenient material for investigations under several aspects for both animals and researchers. Notably, no live trapping or killing are required to obtain samples, as they are collected in the context of the rescue activities and aimed to frame the health status of the animal. The investigated blood samples showed high positive rates to typical roe deer-related microorganisms (such as *Babesia capreoli*), and to the zoonotic agent *Babesia venatorum*. Roe deer were also positive to *Anaplasma phagocytophilum* strains mainly considered apathogenic or limited to wild ungulates. The obtained results underline the importance of a constant investigation on circulating tick-borne pathogens in roe deer, and generally speaking, in wild animal species, due to their potential role as a key factor in the endemic cycle of important infectious agents for domestic and wild animals, as well as humans.

**Abstract:**

*Babesia* ssp. and *Anaplasma* spp. are tick-borne microorganisms representing a possible health risk for domestic and wild animals, as well as humans. Roe deer serve as a suitable reservoir host for some species ascribed to *Babesia* spp. and *Anaplasma phagocytophilum* taxa, also due to its important role in the maintenance of large populations of *Ixodes ricinus*, the main tick vector of these pathogens in Europe. Roe deer populations have been recently expanding throughout Europe, namely in Italy. However, the collection of samples from free-ranging wild animals for diagnostic investigations often includes several practical issues. This problem can be overcome using samples provided by wildlife rescue centers making them available for investigations following routine analyses. The presence of *Babesia* spp. and *Anaplasma* spp. in blood samples of 43 roe deer rescued by a wildlife rescue center in Emilia-Romagna region (Italy) was molecularly investigated. PCR screening revealed the presence of at least one pathogen in 86.05% of the animals, while co-infection occurred in 18.92% of the tested individuals. Zoonotic *Babesia venatorum* was found in 6.98% of the samples, while *Babesia capreoli* and *Anaplasma phagocytophilum* were detected in 74.42% and in 20.93%, respectively. No hematological signs compatible with clinical anaplasmosis or piroplasmosis, as well as absence of intracellular circulating microorganisms in blood smears, were observed, suggesting asymptomatic infection in the tested animals. These results confirm the usefulness of wild rescued animals as convenient source of biological samples for tick-borne pathogens investigation and the role of roe deer as a key factor in the endemic cycle of *Babesia* species and *A. phagocytophilum*.

## 1. Introduction

Over the last decades, increasing interest has been raised towards tick-borne diseases, which are considered to become one of the most important human and veterinary health concerns in the future [1]. In Europe, the most important tick species involved in the transmission of tick-borne pathogens is *Ixodes ricinus*, a hard tick characterized by a triphasic lifecycle and low host specificity, which is able to vehicle a variety of pathogenic microorganisms, such as viruses, bacteria and protozoa [2]. The distribution and abundance of *I. ricinus* is currently expanding both at latitudes and altitudes due to several factors, including climate changes and variable land managing [2,3]. Wild ungulates are considered among the most important hosts for the maintenance of *I. ricinus* populations [4]. In many European countries, as the case of northern Italy, wild ungulates populations are currently expanding, partly due to wildlife management programs and increased number of animals in areas now inhabited by humans [5]. This is the case of roe deer (*Capreolus capreolus*), a species that faced a massive decline at the beginning of the 19th century, due to over-hunting and deforestation, and that is now re-expanding thanks to the abandonment of crops in upland and mountain areas, repopulation programs, hunting regulations and to its high environmental adaptability, with an increased population in Italy of 201.4% from 1984 to 2004 [6,7,8,9]. Roe deer, besides being considered as one of the most important host species for ticks, plays an important role in maintaining several tick-transmitted pathogens, which have raised human and veterinary health concern especially in the last decades [2]. Among these, *Anaplasma phagocytophilum*, whose main vector in Europe is *I. ricinus*, is the etiological agent of tick-borne fever (TBF) in domestic ruminants and granulocytic anaplasmosis in several animal species, humans included [10,11]. Typical hematological signs of *A. phagocytophilum* infection are neutropenia and leukopenia, while clinical signs like weakness, fever, inappetence, constipation, icterus, dehydration, increased infertility have been observed, for example, in cattle and sheep [12,13]. Several studies pointed out that wild ruminants, in particular roe deer, can be the reservoir hosts for this tick-borne agent [14,15]. *Ixodes ricinus* can also vector piroplasms of the genus *Babesia*, a taxon encompassing intra-erythrocytic parasites of the phylum Apicomplexa, with more than 100 ascribed species able to infect domestic and wild animals [16]. Clinical manifestations of babesiosis can vary in severity from asymptomatic to severe and sometimes fatal infections, also depending on the species, and symptoms can include anemia, splenomegaly, hepatomegaly, jaundice; haematological signs include hemolytic anemia and thrombocytopenia [17]. Several *Babesia* species have been reported in roe deer, including two zoonotic agents (*Babesia divergens*, *Babesia venatorum*), and *Babesia capreoli*, currently considered as a specific roe deer parasite [15,17,18].

Due to the role of roe deer as reservoirs of tick-transmitted agents, the screening of these ungulates is crucial to perform studies focused on tick-borne pathogens and evaluate the potential risk for human and animal health. However, samplings from free-ranging wild animals often present several practical issues, such as the time required for sampling, permissions, ethical aspects, high costs, trapping procedures, etc. [19,20]. On the contrary, rescued animals represent a useful and convenient source for biological samples and data from wildlife, without requiring demanding methods of hunting or trapping, that can in turn result in stressful conditions for the animals and need much organization and logistics resources for the researchers. Roe deer populations are increasing in density due to the abandonment of rural areas, changes in human land use, restocking and lack of predators [6,7,21]. Additionally, an increasing geographic expansion of roe deer towards cultivated areas and densely populated and peri-urban territories has been recently reported [7,22,23]. In this context, there is a growing probability of contact between humans and wildlife that in turn intensifies the potential for direct lethal effects (vehicle collisions, predation by dogs, etc.) [24].

Since roe deer are among the most common cervid species in Italy [25], they are frequently recovered by wildlife rescue centers [26]. For these reasons rescued roe deer represent a convenient and easy-to-access source of samples aimed at investigating tick-borne related pathogens.

In this study the presence and co-infection of tick-transmitted microbial agents *A. phagocytophilum* and *Babesia* spp. were investigated in blood samples obtained from wild roe deer rescued by the Piacenza Wildlife Rescue Center (Italy).

## 2. Materials and Methods

### 2.1. Samples Collection

Blood samples from roe deer, of different sexes and ages, rescued in the province of Piacenza (Emilia-Romagna region) during 2019, were obtained from the Piacenza Wildlife Rescue Center (CRAS; Niviano di Rivergano, Italy). Samples were collected in the context of the rescue activities and were aimed to frame the health status of the animal before taking any medical decision; the leftover part of the blood samples were used for the purpose of the present research. According to the guidelines of the authors’ institution, a formal approval of the Ethical Committee was not required (EC decision 29 October 2012, renewed with the protocol no. 02-2016). The reasons for the admission varied (e.g., car road accident, trauma due to combine harvesters or other causes like predation or imprinting). Blood was withdrawn from the cephalic vein within 2 h of arrival to the center, and put in EDTA-coated tubes for complete blood cell analysis. Blood smears were obtained immediately, stained with May Grünwald-Giemsa and examined under a light microscope (magnification 100×). The leftover part of blood samples was destined to molecular analyses and stored at −80 °C until use.

### 2.2. Molecular Analyses

DNA was isolated from 200 μL of whole blood using the DNeasy Blood and Tissue Kit (Qiagen, Hilden, Germany) following the manufacturer’s instructions. Extracted DNA samples were quantified using a spectrophotometer (Nanodrop ND 1000, Thermo Scientific, Wilmington, DE, USA) and stored at −80 °C until further analyses. Quality of the DNA extraction was assessed by amplifying a fragment of the 12S rDNA gene of *C. capreolus* [27].

All samples were screened for the presence of *Babesia* spp. DNA using a qualitative PCR targeting a 411−452 bp region of the 18S rDNA gene [28]; the presence of *Anaplasma* spp. was assessed using a qualitative PCR targeting a ~490 bp region of the 16S rDNA gene and which detects *Ehrlichia* and *Anaplasma* species [29]. PCR products were loaded on agarose gel, bands corresponding to the positive amplicons were excised and purified with Wizard^®^ SV Gel and PCR Clean-Up System Kit (Promega, Madison, WI, USA) following the manufacturer’s instructions, and subsequently bidirectionally Sanger sequenced. The obtained sequences were manually curated, assembled using SeaView 4.7 (PRABI-Doua, Lyon, France) [30], and deposited in GenBank. After NCBI Blast comparison of the obtained sequences with those available in GenBank, unresolved *B. divergens*/*B. capreoli* positive samples were subjected to a PCR protocol described elsewhere [31] to evaluate the presence of the three-base differences in the 18S rDNA that can discriminate the two species [17].

### 2.3. Phylogenetic and Statistical Analyses

Phylogenetic inference was performed using Maximum Likelihood method and Kimura 2-parameter model [32] chosen according to the BIC criterion and conducted with Mega X software version 10.1.8 (MEGA, Pennsylvania, PA, USA) [33].

Prevalence was calculated with confidence intervals at 95%. Statistical significance between positivity of males and females to *Babesia* spp., *Anaplasma* spp., and co-infections, was inferred using Fisher’s exact test; the association between the presence of *Babesia* spp. and *Anaplasma* spp. was analyzed using Chi-squared test. The online tool “Social Science Statistics^®^” Calculator was used for statistical inference (www.socscistatistics.com; accessed on 26 October 2021) [34].

## 3. Results

A total of 43 blood samples from as many rescued roe deer individuals (21 females and 22 males) were collected by the Piacenza Wildlife Rescue Center. Complete blood cell count did not show hematological signs compatible with clinical anaplasmosis (leukopenia and neutropenia) or piroplasmosis (hemolytic anemia) (data not shown). The main hematological abnormalities found in were mainly attributable to the cause of the recovery (blood loss anemia) or to the stress due to the capture and pain (neutrophilia and lymphopenia). Blood smears observation showed no parasitic inclusions in any analyzed sample.

All the extracted DNA samples tested for roe deer 12S rDNA gene showed bright PCR bands when run in agarose gel, suggesting good DNA quality and absence of PCR inhibition. Overall, 37 out of 43 analyzed DNA samples (86.05%; 95% CI: 75.69–96.40) resulted positive to at least one pathogen and 7 of them (18.92%; 95% CI: 6.30–31.54) showed simultaneous infection with the two investigated microbial agents. In detail, 35 samples (81.4%; 95% CI: 69.76–93.03) resulted positive to *Babesia* spp., and 9 samples (20.93%; 95% CI: 8.77–33.09) were positive to *Anaplasma* spp.

All the obtained *Babesia* spp. 18S rDNA amplicons were sequenced, and 32 out of 35 sequences showed 100% unresolved identity with *B. divergens*/*B. capreoli*. Additional PCR analyses revealed 100% identity with *B. capreoli* sequence FJ944827 (showing an overall prevalence of 74.42%; 95% CI: 57.28-91.56). Three samples (overall prevalence 6.98%; 95% CI: 0.00–16.98) showed 100% identity with several *B. venatorum* sequences present in GenBank (e.g., MG344777). The obtained *Babesia* spp. sequences were deposited in GenBank under the accession numbers OK598971-OK598972. No *Babesia* mixed infections were observed in the same sample.

**Figure 1:**
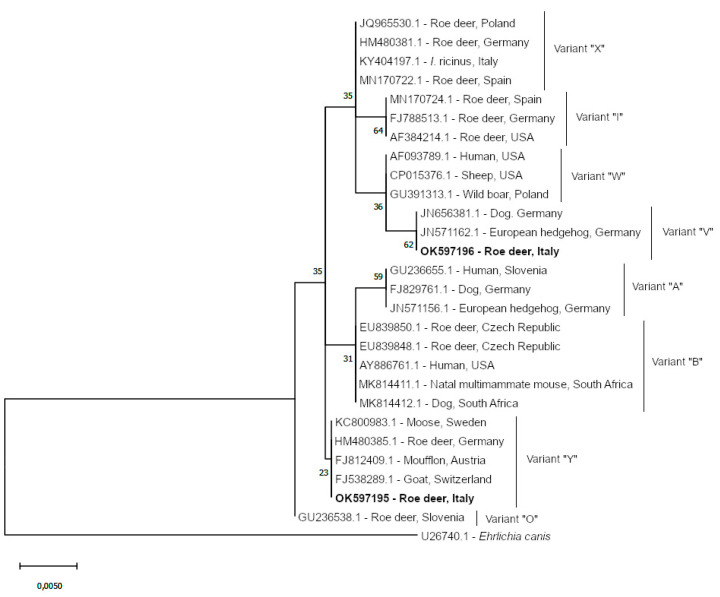
Maximum likelihood phylogenomic tree of the partial 16S rDNA gene of *A. phagocytophilum* obtained with MEGA X [33] and Kimura 2-parameter [32] model showing clustering variants (100 replicates; bootstrap values are indicated at the nodes). *Ehrlichia canis* 16S rDNA sequence (U26740) was used as outgroup. The tree is drawn to scale, with branch lengths measured in the number of substitutions per site. Isolates identified in this study are indicated in bold font.

Sequencing of the nine positive *Anaplasma* spp. amplicons showed identity with *A. phagocytophilum*, and the subsequent phylogenetic analysis highlighted the presence of two sequence variants (Figure 1). One isolate (accession OK597195; two positive samples) clustered with *A. phagocytophilum* variant “V” group; the second isolate (accession OK597196; seven positive samples) clustered with variant “Y” group [15,35].

No significant differences were observed in infection rates between females and males regarding the investigated pathogens or co-infections (*p* > 0.05), but a significant association between the two pathogens in the infected hosts was observed (*p* < 0.05).

## 4. Discussion

In this study, the presence of *Babesia* spp. and *Anaplasma* spp. in rescued roe deer was investigated. The overall prevalence of *Babesia* spp. in roe deer (81.4%) is comparable to other studies performed in Italy, specifically in areas close to the study site [36]. Additionally, the obtained results are in line with surveys performed in other European countries [37,38,39,40]. *B. capreoli* was the most common detected *Babesia* species. It must be noted that *B. divergens* is widely overestimated in wildlife due to its erroneous overlap with *B. capreoli* [41,42]. For this reason, accurate molecular analyses aimed to the discrimination of *B. divergens/B. capreoli* need to be performed for a precise evaluation of human and animal health risk related to the presence of these two piroplasms. The two species are serologically indistinguishable and require sequencing analyses of the 18S rDNA gene amplicons for the identification of the three-nucleotide variation that characterizes each species [17]. A previous study performed on roe deer in a close area revealed the presence of *B. divergens*-related sequences, clustering with the single *B. capreoli* gene sequence (AY726009) available at that time [36]. However, the mentioned survey was prior to the redescription of *B. capreoli* [17,43], and no related 18S rDNA gene entries, systematically able to differentiate *B. divergens* and *B. capreoli*, were available for a precise classification [17]. In the light of the three-nucleotide variation described between the two considered species, previous results concerning *B. divergens*-like findings in the study area should be reconsidered to obtain a clear epidemiological picture of *Babesia* species in the area. Despite the apparently lack of pathogenicity for human and domestic animals, *B. capreoli* appears as a possible concern for some wildlife species, occasionally causing disease in wild ungulates [38]. Deeper investigations on babesiosis in wildlife are needed, since limited information on few wild species are currently available [38,44]. Clinical babesiosis is commonly observed to be rare in free-ranging wild ruminants, and asymptomatic babesiosis seems to be the most widespread condition [45]. However, several studies reported cases of clinical signs of babesiosis in roe deer [38], with symptoms that included high parasitemia prevalence [17]. In the present study, despite the high prevalence of *B. capreoli* in the tested animals, hematological signs compatible with a possible clinical disease were not found in any of the cases. This result hints that the tested roe deer were healthy carriers of *B. capreoli*, and that infection might be endemic rather than emerging. In addition, the hypothesis of the role of roe deer as reservoir host for *B. capreoli*, with asymptomatic infections that rarely induce clinical symptoms, is supported. Since wildlife is recognized for its role in the transmission of pathogens to livestock, and ungulates share many pathogens with cattle, beyond acting as possible reservoirs and sustaining tick populations [46], attention should be paid to those areas with high *B. capreoli*-positive rates. Indeed, domestic animals, such as sheep, have been reported as hosts for *B. capreoli* [42]. On the contrary, *B. venatorum*, which has been previously identified in ticks and roe deer in neighboring areas [31,36,47], showed prevalence rates in accordance with former published data [36]. Infection with *B. venatorum*, whose primary host is considered to be roe deer, has been reported in several human cases in Europe and China, originally in immunocompromised patients, and, more recently, also in immunocompetent ones [48,49]. Further investigations on this zoonotic agent should be carried out to evaluate the actual health risk for humans, domestic and wild animals. Furthermore, a previous study reported the presence of *B. venatorum* more often in roe deer individuals found dead, suggesting this *Babesia* species as an additional cause of death [14,50]. However, hematological parameters of the *B. venatorum*-positive individuals did not show any clinical sign of infection.

*Anaplasma phagocytophilum* was detected in roe deer with a prevalence of 20.93%, in line with prevalence rates found in other European countries, ranging from 9.6% to 100% [14,39,40,51]. In particular, several studies performed on spleen have provided very high prevalence rates as already stated elsewhere [14,51]; for this reason the observed prevalence in the study area could be underestimated. Unfortunately, due to the type of recruitment of roe deer (rescued animals) only blood was obtained, and no information about the possible presence of the parasite in other tissues may be obtained. Additionally, prevalence of *A. phagocytophilum* in other Italian regions showed higher prevalence rates in different wild ungulates [52,53,54], but the use of different molecular approaches could have influenced the outcoming results. Roe deer is considered to play an important role in spreading *A. phagocytophilum* [15,35], as it is also considered one of the major actors in the maintenance of *I. ricinus* populations [55,56]. Transovarial transmission of *A. phagocytophilum* has been described as absent or inefficient in *I. ricinus* [57], hence the role of reservoir hosts, as the case of roe deer, which are able to maintain large tick populations, becomes crucial for the spread of anaplasmosis [51]. Roe deer is able to migrate easily for long distances while carrying variable tick loads, thus promoting the spreading of ticks in several areas [15] and, in turn, enhancing the spreading of potential zoonotic agents. Different gene targets have been employed in determining variants and strains of *A. phagocytophilum*, such as *ankA*, *msp4*, *groEL* and 16S rDNA [40,58]. Compared to other genes, the 16S rDNA represents a more conserved genetic region with a limited genetic variability, thus resulting uninformative for precise identification of genetic variants and for host association inferences [35,39]. Although the 16S rDNA gene is not considered a good discriminatory marker for *A. phagocytophilum* [59], some authors reported it as a useful starting point for the discrimination of variants causing granulocytic anaplasmosis from non-pathogenic ones [35]. The most frequent sequence variant observed in this study (seven out of nine positives) clustered with sequences belonging to *A. phagocytophilum* variant “Y”, which has been previously related to wild ruminants, mainly cervids. It is one of the two most widespread variants in Europe and is currently considered apathogenic [35,39]. The less prevalent *A. phagocytophilum* sequence variant identified in this work clustered with variant “V” which has been classified as potentially specific for wild ruminant, and was detected previously in roe deer, and also in dogs [15,39]. Taking into account the results concerning the isolates detected in this study, attention should be paid to roe deer as contributors to the endemic cycle of possible pathogenic variants of *A. phagocytophilum* for wild ungulates and, potentially, domestic animals. However, further analyses should be performed using additional molecular markers other than 16S rDNA, in order to deeper investigate the *A. phagocytophilum* variants circulating in the area and precisely characterize those that could represent a health threat for animals and humans.

Overall, no gender-dependent infection rates were observed for both pathogens, as well as for co-infections, supporting similar data for roe deer previously reported [39,50]. In this study, 18.92% of the tested individuals showed co-infection of *Babesia* spp. and *A. phagocytophilum*, which is significantly lower compared to other surveys performed in roe deer in Europe [15,39,40,60]. Co-infection of the two pathogens in wild ungulates has been matter of investigations in the recent years for their possible interactions in the mammalian host [41]. Even though the potential synergic role between the two pathogens needs to be unraveled, several studies have already pointed out the generalized immunosuppressive activity of *A. phagocytophilum* in the host, which affects the number and functions of lymphocytes and granulocytes in peripheral blood. Thus, infection with *A. phagocytophilum* could put the animal at risk for increased susceptibility of infection by various *Babesia* species [11,51,61]. Microbial co-infections in vertebrate hosts, as well as in tick vectors, require growing attention since synergic and antagonistic interactions could in turn promote, worsen or modify the onset and clinical signs of diseases caused by a single pathogen.

As already stated, despite the high prevalence of the investigated pathogens, no hematological signs were compatible with a possible clinical disease. Blood smears and results of complete blood cell count showed no presence of any circulating microorganism, suggesting a transient and limited parasitemia. The absence of *Babesia* cells in blood smears was already observed in previous surveys, but parasites were eventually detected in blood following few days of autologous cell culture [62], hinting that more sensitive techniques (as the case of molecular analyses) are required for the detection. Hematological parameters highlighted the absence of possible pathologies and indicated the presence of an asymptomatic infection. Nonetheless, it is not excludible that positivity to these tick-borne pathogens could have played a risk factor for roe deer in being more subjected to car accidents, predation, or traumas.

## 5. Conclusions

In summary, this study highlights the valuable contribution of rescued animals as a source of samples for investigations on tick-borne and zoonotic pathogens. Wildlife rescue centers represent precious and convenient sources of material to evaluate the current health status and condition of the wildlife populations, requiring a minimal sampling effort for both animals and researchers. However, the usefulness of this data can mainly be attributed to those species which are more widespread and thus more commonly rescued by these centers, as the case of roe deer in Europe.

This work also confirms roe deer as one of the main possible reservoir hosts for infectious agents that can affect wildlife, as the case of *B. capreoli*. Hematological data of wildlife (namely roe deer) in relation to *Babesia* spp. and *A. phagocytophilum* are limited, and this field should be deeper investigated for a better comprehension of the infection mechanisms in wild animals. In addition, roe deer has been confirmed as reservoir host for zoonotic agents (or potential ones) as the case of *B. venatorum*. Co-infection between *Babesia* spp. and *A. phagocytophilum* requires further investigations to understand which of the two pathogens could influence the infection of the other and in which way. The current expansion of roe deer populations throughout Europe, that in turn results in the possible increasing coexistence between deer and humans and/or domestic animals, dictates an adequate knowledge of the potential infectious diseases carried by free-ranging wild ungulates.

## Data Availability

The nucleotide sequences generated in this study from *Babesia* spp. partial 18S rDNA gene (OK598971-OK598982) and *Anaplasma phagocytophilum* partial 16S rDNA gene (OK597195-OK597196) were deposited in GenBank (NCBI).
